# AutoClickChem: Click Chemistry *in Silico*

**DOI:** 10.1371/journal.pcbi.1002397

**Published:** 2012-03-15

**Authors:** Jacob D. Durrant, J. Andrew McCammon

**Affiliations:** 1 Department of Chemistry & Biochemistry, University of California San Diego, La Jolla, California, United States of America; 2 Department of Chemistry & Biochemistry, NSF Center for Theoretical Biological Physics, National Biomedical Computation Resource, University of California San Diego, La Jolla, California, United States of America; 3 Department of Pharmacology, University of California San Diego, La Jolla, California, United States of America; 4 Howard Hughes Medical Institute, University of California San Diego, La Jolla, California, United States of America; National Evolutionary Synthesis Center, United States of America

## Abstract

Academic researchers and many in industry often lack the financial resources available to scientists working in “big pharma.” High costs include those associated with high-throughput screening and chemical synthesis. In order to address these challenges, many researchers have in part turned to alternate methodologies. Virtual screening, for example, often substitutes for high-throughput screening, and click chemistry ensures that chemical synthesis is fast, cheap, and comparatively easy. Though both *in silico* screening and click chemistry seek to make drug discovery more feasible, it is not yet routine to couple these two methodologies. We here present a novel computer algorithm, called AutoClickChem, capable of performing many click-chemistry reactions *in silico*. AutoClickChem can be used to produce large combinatorial libraries of compound models for use in virtual screens. As the compounds of these libraries are constructed according to the reactions of click chemistry, they can be easily synthesized for subsequent testing in biochemical assays. Additionally, *in silico* modeling of click-chemistry products may prove useful in rational drug design and drug optimization. AutoClickChem is based on the *pymolecule* toolbox, a framework that may facilitate the development of future python-based programs that require the manipulation of molecular models. Both the *pymolecule* toolbox and AutoClickChem are released under the GNU General Public License version 3 and are available for download from http://autoclickchem.ucsd.edu.

This is a *PLoS Computational Biology* Software Article

## Introduction

Though the pharmaceutical industry has been the traditional steward of drug development, in recent years academic institutions have played an increasingly important role as well. Formal academic drug-discovery centers established at universities in Belgium, Sweden, the United Kingdom, and the United States have already made great contributions towards the development of novel treatments for neglected and orphan diseases, projects that are generally not financially appealing to industry [1]. Academia may be particularly well suited for the earliest stages of drug discovery, such as target and lead identification [2]. Fruitful collaborations between academia and industry are also becoming more commonplace.

Despite their growing interest in drug discovery, academic researchers, as well as some in industry, often lack the financial resources available to scientists working in “big pharma.” High costs include those associated with high-throughput screening and chemical synthesis. Fortunately, limited financial resources have spurred innovation. Virtual screening, a computational technique that can, in part, mimic high-throughput screening *in silico*, is one example of this kind of innovation. Traditionally, high-throughput biochemical screens have constituted and continue to constitute a critical but expensive step in the earliest stages of drug development. Vast and costly libraries of chemical compounds, often in excess of 100,000 molecules, are screened against identified targets of known pharmacological importance in an attempt to identify potent ligands. Robotics and miniaturized/parallelized biochemical assays make such large-scale screening efforts possible. However, with some notable exceptions, the high cost and man-power demands of high-throughput screens make them inaccessible to many researchers.

Virtual screening aims to make high-throughput projects more feasible. Computer docking programs attempt to position candidate ligands within the binding pockets of crystallographic, NMR, or theoretical protein structures in order to predict binding affinity. While docking programs are powerful tools, they do have shortcomings that limit applicability [3], [4]. The programs depend on accurate, atomistic, small-molecule and receptor models (including important bound waters) that can be laborious to prepare; they employ scoring functions that are optimized for speed at the expense of accuracy, often making it difficult to distinguish between nanomolar and micromolar inhibitors; and they often ignore aspects of molecular flexibility that doubtless play important roles in receptor-ligand binding.

Consequently, docking algorithms are not yet accurate enough to assess the binding of a single ligand with certainty, but they can in many circumstances be used to enrich a pool of candidate ligands for true binders [3], [5], [6]. The compounds of this enriched pool of potential ligands, in number far fewer than the total number of compounds in the original library, are then experimentally validated to identify true binders. Virtual screening methodologies have already been used to identify many ligands [7], [8]. A few examples include inhibitors of *Trypanosoma brucei* RNA editing ligase 1 [9], [10], *Trypanosoma brucei* UDP-galactose 4′-epimerase [11], and *Homo sapiens* stromelysin-1 [12].

The high costs associated with high-throughput screens are not the only impediments to drug design. Chemical synthesis can also be very costly and time consuming. The libraries of hundreds of thousands of compounds required for high-throughput screens are expensive to synthesize and/or to purchase commercially. Additionally, following the identification of true ligands, drug optimization requires chemical synthesis in order to improve potency and other pharmacological and toxicological properties.

Dr. Barry Sharpless recently proposed a new chemistry paradigm called “click chemistry” [13] that can help overcome the financial impediments associated with chemical synthesis. There are approximately 10^60^ possible drug-like compounds [2]. Any hopes of thoroughly exploring so large a chemical space must be abandoned from the outset. Given that only an infinitesimally small portion of all possible molecules can ever be synthesized, the chemical reactions used to synthesize potential ligands might as well be limited to those reactions that are ideal; only “click” reactions that are comparatively easy to perform, safe, and cheap need be considered [14]. Using these ideal click-chemistry reactions, academic researchers have produced inhibitors of α-1,3-fucosyltransferase [15], HIV protease [16], acetylcholine esterase [17], [18], [19], carbonic anhydrase II [20], influenza neuraminidase [21], and protein tyrosine phosphatase 1B [22].

Both virtual screening and click chemistry have, in part, the same objective: to make drug discovery practical even when financial resources are limited. Given their philosophical similarities, it is curious that these two methods have not been coupled. We here present a novel algorithm called AutoClickChem that can simulate many click-chemistry reactions *in silico*. Like some other freely available [23], [24], [25] and commercial software packages (e.g., CambridgeSoft's ChemOffice Ultra [26], Tripos' CombiLibMaker [27], [28], ChemAxon's Reactor [29], etc.), AutoClickChem can be used to generate combinatorial libraries for virtual screening. However, AutoClickChem is unique in that it simultaneously satisfies the following criteria: 1) the program is freely available under an open-source license; 2) a web-server application has been implemented that permits use without requiring installation; 3) the generated compounds can be easily synthesized for subsequent testing in biochemical assays because they are constructed according to the reactions of click chemistry; 4) there is no need to specify linker atoms *a priori* because reacting functional groups are automatically detected; and 5) all structures are automatically generated in three dimensions (Table 1). Additionally, AutoClickChem is based on the *pymolecule* toolbox, a framework that may facilitate the development of other python-based programs that require the manipulation of molecular models.

**Table 1:** A comparison of several computer programs for virtual combinatorial-library generation.

	Reference	Free	Open Source	Server Application	Synthesizability of Products	Auto-Identification of Reactive Atoms/Groups	3D Products Produced
AutoClickChem^1^		**+**	**+**	**+**	**+** (click chemistry)	**+**	**+**
SmiLib^2^	[26]	**+**	**+**	**−**	−	−	−
SLF_Libmaker^3^	[24]	−	−	−	−	−	?
ChemOffice Ultra^4^	[26]	−	−	−	−	−	−
CombiLibMaker^5^	[27], [28]	−	−	−	?	?	**+**
ChemAxon Reactor^6^	[29]	**+** (for academics only)	−	**+** (restricted)	**+** (user-specified reactions)	**+**	−

1. autoclickchem.ucsd.edu.

2. gecco.org.chemie.uni-frankfurt.de/smilib/.

3. www.idealp-pharma.com.

4. cambridgesoft.com.

5. tripos.com.

6. chemaxon.com.

“Free” means the software is available free of charge, “Open Source” means the source code can be freely modified, “Server Application” means the software is available for use remotely over the internet (without installation), “Synthesizability of Products” means the software takes into account actual chemical reactions when generating compounds *in silico*, “Auto-Identification of Reactive Atoms/Groups” means the program automatically identifies reactive atoms or chemical groups so that the user need not manually annotate, and “3D Products Produced” means the program automatically generates models with 3D coordinates.

### Design and Implementation

#### AutoClickChem

As input, AutoClickChem accepts PDB models of two small molecules, the two desired reactants. The program begins by automatically identifying functional groups such as alkynes, azides, and epoxides that are known to participate in any of a number of predefined chemical reactions, described in detail Text S1. Once the relevant functional groups have been identified, the program determines which reactions are possible and begins to assemble models of the appropriate products.

The steps required to assemble the products associated with each predefined chemical reaction are unique. As AutoClickChem has been implemented in python and is open source, interested readers can examine the source code to determine how each reaction is programmed. Additional details can also be found in Text S1. To illustrate the general procedure, we here describe how AutoClickChem mimics the azide-alkyne Huisgen cycloaddition, a representative reaction that has been called the “cream of the crop” of click chemistry [13].

**Figure 1:**
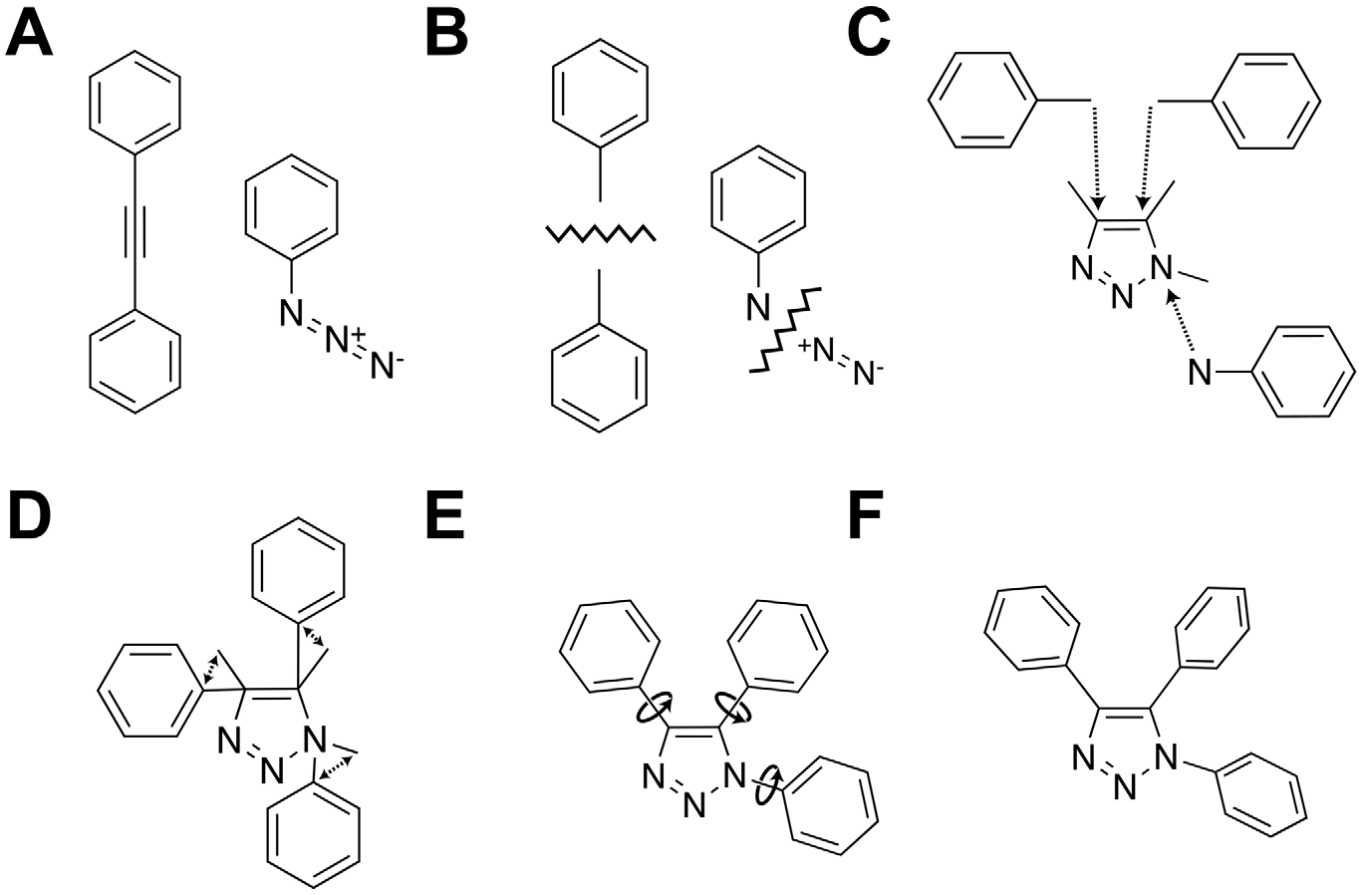
A schematic showing how AutoClickChem mimics the azide-alkyne Huisgen cycloaddition. A) This cycloaddition combines an alkyne and an azide into a 1,2,3-triazole product. B) As a first step, AutoClickChem fragments the alkyne along its triple bond and the azide along the bond connecting its proximal and medial azide nitrogen atom. C) The fragments are then translated so that atomic “handles” are superimposed on top of the corresponding atoms of a 1,2,3-triazole model. D) Next, the fragments are rotated about the handle atoms in order to minimize the distance between the handle-adjacent atoms and the corresponding atoms on the 1,2,3-triazole model. E) The positioned fragments are then rotated in order to reduce steric hindrance. F) Finally, redundant atoms are deleted, and the fragment and 1,2,3-triazole model atoms are merged into a single final structure.

The azide-alkyne Huisgen cycloaddition combines an alkyne and an azide (Figure 1A) into a 1,2,3-triazole product. As a first step, AutoClickChem fragments the alkyne along its triple bond and the azide along the bond connecting its proximal and medial azide nitrogen atoms (Figure 1B). Note that the resulting fragments have atomic “handles” comprised of what were the alkyne carbon atoms and the proximal azide nitrogen atom. The fragments are then translated so that these handles are superimposed on top of the corresponding atoms of a 1,2,3-triazole model (Figure 1C). Next, the fragments are rotated about the handle atoms in order to minimize the distance between the handle-adjacent atoms and the corresponding atoms on the 1,2,3-triazole model (Figure 1D). The positioned fragments are then rotated in order to reduce steric hindrance (Figure 1E). Finally, redundant atoms are deleted, and the fragment and 1,2,3-triazole model atoms are merged into a single final structure (Figure 1F). For non-symmetric alkynes, AutoClickChem generates both regioisomers.

#### The *pymolecule* toolbox

AutoClickChem is based in part on the open-source *pymolecule* toolbox, a framework that facilitates the manipulation of molecular models. We have used beta versions of this toolbox to develop a number of other applications, including HBonanza [30], BINANA [31], POVME [32], and NNScore [33]. With AutoClickChem, the *pymolecule* toolbox has matured. All supporting functions are now contained within a single python file (pymolecule.py) that can be easily included in other projects. Additionally, full documentation is available describing each *pymolecule* definition.

The *pymolecule* toolbox contains three python classes: Point, Atom, and Molecule. The Point class is used to create and manipulate objects with three coordinates, *x*, *y*, and *z*, be they points or vectors in three-dimensional space, and the Atom class stores and manipulates atomic information. The details of these classes are well documented in the source code.

However, the Molecule class, a useful class for manipulating entire molecular structures, merits a more detailed description because it is likely the class that will be most frequently accessed by those developing *pymolecule*-based applications. First, the Molecule class contains two python definitions, *load_pdb* and *save_pdb*, for loading and saving PDB information from/to files.

Six additional Molecule definitions can be used to manipulate the atomic coordinates of a molecular model. Two definitions are used for model translation: *translate_molecule* translates all atomic coordinates by a specified vector, and *set_atom_location* translates all atomic coordinates such that a specified atom resides at a desired coordinate. Three additional definitions rotate the molecular model: *rotate_molecule_around_pivot* rotates all atomic coordinates about a specified point, and *rotate_molecule_around_a_line* and *rotate_molecule_around_a_line_use_atom_indicies* rotate all atomic coordinates about a line segment defined by two terminal Point objects or by the coordinates of two Molecule atoms, respectively. Finally, the *align_another_molecule_to_this_one* definition aligns a second molecule (*molecule_to_align*) to the current one. “Tethers” are defined connecting pairs of atoms, where each of the constituent atoms belong to a separate molecular model. The *molecule_to_align* model is then translated and rotated as necessary to minimize the summed length of the defined tethers.

Several definitions return information about bond connectivity. The *number_of_neighors_of_element* definition counts the total number of atoms of a specified element bound to an atom of interest; *index_of_neighbor_of_element* considers all the atoms bound to a specified atom and returns the index of the first atom of the specified element; *hybridization* determines the orbital hybridization of a specified atom, based in large part on its connectivity; *in_same_ring* determines if two specified atoms are contained in the same ring system; and *get_branch* partitions a molecular model into two by essentially “cutting” along a specified bond.

Finally, two definitions are used to manipulate multiple Molecule objects. The *merge_with_another_molecule* definition merges a second Molecule object with the current one, and the *distance_to_another_molecule* function calculates the minimum distance between the atoms of the current Molecule object and a second one.

Examples illustrating how the *pymolecule* toolbox is used to simulate click-chemistry reactions *in silico* can be found in Text S1.

## Results

We here present a novel computer algorithm, called AutoClickChem, capable of performing click-chemistry reactions *in silico*. AutoClickChem can be used to produce large combinatorial libraries of compound models for use in virtual screens. As the compounds of these libraries are constructed according to the reactions of click chemistry, predicted ligands can be easily synthesized for subsequent testing in biochemical assays. AutoClickChem is based in part on the *pymolecule* toolbox, an open-source framework that may facilitate the creation of other python-based applications requiring the manipulation of molecular models.

### Click Chemistry Reactions

Though the azide-alkyne Huisgen cycloaddition [34] is the quintessential click-chemistry reaction, there are in fact many reactions with high chemical yields, inoffensive byproducts, simple reaction conditions, and physiologically stable/easily purified products [13], [14]. A description of the “click” reactions that AutoClickChem can simulate *in silico* is given in Text S1; a useful summarizing graphic is also provided (Figure S1).

By generating molecular models based on the reactions of click chemistry, AutoClickChem facilitates interactions between computational and synthetic chemists. When pursuing *de-novo* drug-design projects, many computational chemists (ourselves included!) are notorious for generating compounds that, while predicted to be potent, are nevertheless difficult to synthesize. AutoClickChem helps computational chemists stay within the realm of synthesizability, thus facilitating the transition from *in silico* to *ex silico* testing.

### Generating a Virtual Library of Easily Synthesizable Compounds

To demonstrate how AutoClickChem can be used to generate a large virtual library of easily synthesizable compound models for virtual-screening projects, we constructed a library from models of compounds available commercially through hit2lead.com. In all, 939 suitable alkyne models and 1,220 suitable bromide models were ultimately generated from selected hit2lead compounds. AutoClickChem was first used to convert the 1,220 bromides into 1,215 azides. Next, these azide products were reacted with the 939 alkynes *in silico* to produce 2,281,770 1,2,3-triazole products. Any of these products could in theory be easily synthesized *in vitro via* the azide-alkyne Huisgen cycloaddition reaction [34]. When only those models that satisfied all of Lipinski's rule-of-five criteria were considered [35], approximately 800,000 drug-like models remained. Additional details describing the generation of this virtual library can be found in Text S1.

When creating large virtual libraries, the ability to generate products in three dimensions is particularly useful. While programs certainly do exist for converting dimensionless molecular representations (*e.g.*, SMILES strings) into 3D structures, converting hundreds of thousands of models is computationally intensive. With AutoClickChem, this extra step is unnecessary.

To demonstrate the diversity of the compounds generated, we randomly selected fifty azide and fifty alkyne models from the libraries described above. OpenBabel [36] was subsequently used to characterize the corresponding 1,2,3-triazole products according to molecular weight, the number of atoms, the partition coefficient (logP), the polar surface area, and the molar refractivity (Table 2). This characterization confirmed that the compounds are diverse despite having been generated from a limited set of reactants.

**Table 2:** To demonstrate the diversity of the compounds generated, fifty azides and fifty alkynes were selected at random and reacted *in silico* using AutoClickChem.

	Molecular Weight	Number of Atoms	logP	PSA	MR
Minimum	395.5	41	0.9	69.0	103.3
Maximum	593.6	92	6.5	219.0	168.8
Mean ± Stan. Dev.	502.8±29.2	74.6±9.6	3.8±1.1	117.0±23.5	146.4±13.5

“logP” refers to the estimated partition coefficient, “PSA” refers to the polar surface area, and “MR” refers to the molar refractivity.

Though we recommend creating custom libraries specifically designed for target proteins of interest, this large, diverse virtual library may nevertheless serve as a useful starting point for any virtual-screening project. A fast docking program like AutoDock Vina [37] running on a 100-processor cluster should be able to screen the whole library against a single protein structure in a matter of days. The AutoClickChem-generated virtual library herein described is freely available for download in several formats on the AutoClickChem website at http://autoclickchem.ucsd.edu.

### Optimization of Tacrine, a Known Acetylcholinesterase Inhibitor

Having demonstrated how AutoClickChem can be used to generate a large virtual library of easily synthesizable compound models, we next show how the program can be used for ligand optimization. To this end, we replicate *in silico* a recent study conducted by Krasinski et al. [18] that sought to optimize the binding affinity of tacrine, a known inhibitor of acetylcholinesterase (AChE). AChE inhibitors are among the approved pharmacological treatments of Alzheimer's disease, myasthenia gravis, and glaucoma. Krasinski et al. started by creating an azide analogue of tacrine. This azide was then mixed in the presence of the enzyme with 23 acetylene reagents not known to bind AChE. Remarkably, of the 46 possible 1,2,3-triazole products, only two formed *in situ*. These two ligands were subsequently identified by HPLC-mass spectrometry. The *syn* compounds (R)-TZ2PIQ-A5, TZ2PIQ-A6, and (S)-TZ2PIQ-A5 were ultimately found to inhibit mouse AChE with K_d_ values of 100, 410, and 500 fM, respectively.

To replicate this study *in silico*, AutoClickChem was used to generate the same 46 compounds synthesized by Krasinski et al. When alternate charged, tautomeric, ring-conformational, and stereoisomeric states were considered, 1,416 small-molecule models were ultimately produced. These were docked into a crystal structure of mouse AChE (PDB ID: 1Q83) [38] using AutoDock Vina [37], and subsequently rescored with the AutoDock 4.0 scoring function [39], without redocking. Details describing the docking protocol used can be found in Text S1.

AutoDock predicted that the binding affinities of the *syn* compounds (R)-TZ2PIQ-A5, TZ2PIQ-A6, and (S)-TZ2PIQ-A5, the three most potent inhibitors, would be −17.56, −18.43, and −17.74 kcal/mol, respectively. Remarkably, these three compounds were among the four best ranked compounds of the virtual screen. Additionally, compounds in the *syn* conformation tended to be favored, in harmony with experiment.

### Optimization of Analogues of a Known Protein Tyrosine Phosphatase 1B Inhibitor

As a second demonstration of drug optimization, AutoClickChem was used to replicate a recent study conducted by Srinivasan et al. [22] wherein analogues of a known protein tyrosine phosphatase 1B (PTP1B) inhibitor, a potential treatment for type 2 diabetes, were optimized to improve binding affinity. Srinivasan et al. began by attaching alkynes to 5 of the analogues. Additionally, 14 aromatic azides were synthesized that were thought likely to bind to a nearby secondary site. Copper (I) was used to catalyze the azide-alkyne Huisgen cycloaddition so that only the 1,4 regioisomers were produced [40]. Of the roughly 70 1,2,3-triazole compounds synthesized, one, called A13, was particularly potent, with an IC_50_ of 4.7 µM against PTP1B.

To replicate this study *in silico*, we used AutoClickChem to generate the same 70 compounds. When alternate charged, tautomeric, ring-conformational, and stereoisomeric states were considered, there were 108 small-molecule models. These were docked into a crystal structure of PTP1B (PDB ID: 2F71) [41] using AutoDock Vina [37], and subsequently rescored with the AutoDock scoring function [39], without redocking. The best inhibitor identified experimentally ranked 5^th^ in our virtual screen, placing it in the top 5% of all models docked.

**Figure 2:**
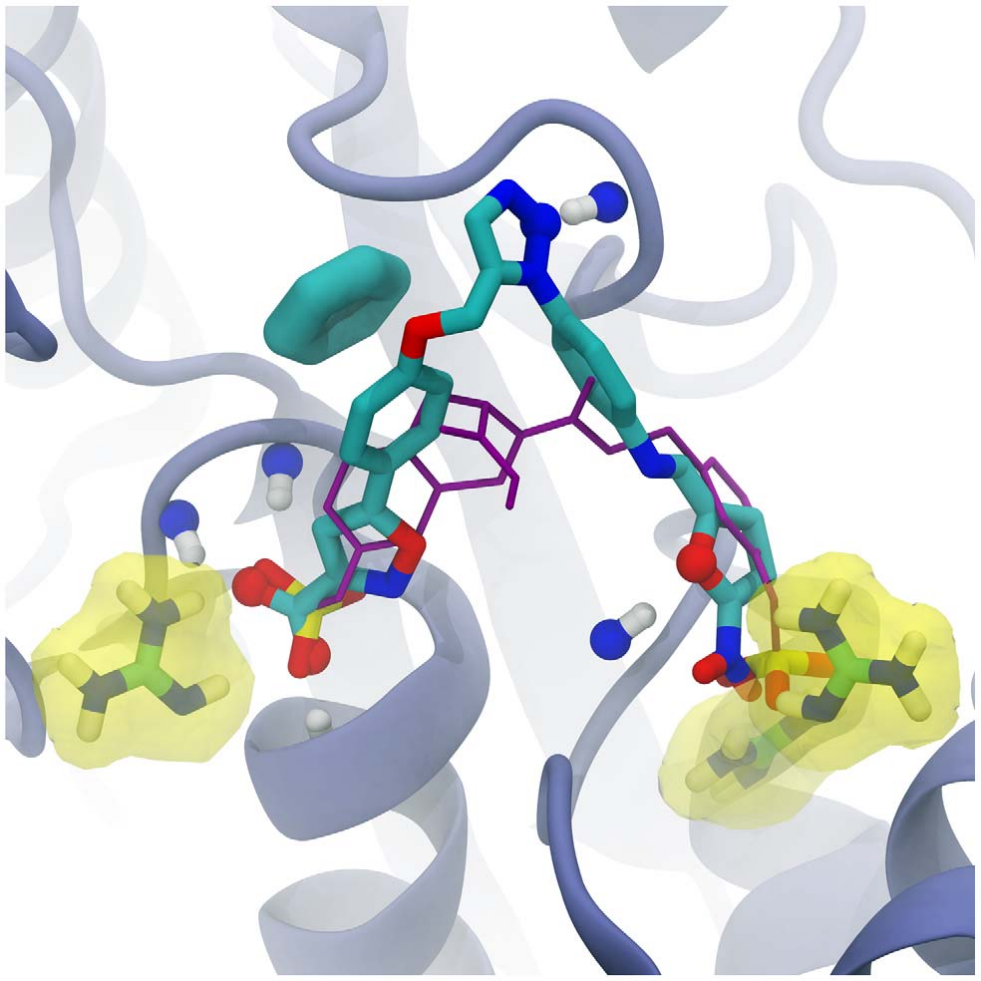
The top-scoring predicted PTP1B ligand (in licorice representation), docked into the receptor active site. Protein residues that participate in electrostatic interactions are highlighted in yellow. Atoms that participate in receptor-ligand hydrogen bonds are shown in ball-and-stick representation. The aromatic ring of the receptor tyrosine residue that participates in π-π stacking and T-stacking interactions with the ligand is shown in thick licorice representation. The crystallographic pose of a known inhibitor is shown in purple, with key sulfonate moieties shown colored by element in licorice representation. Portions of the protein have been removed to facilitate visualization.

As the inhibitors identified by Srinivasan et al. [22] were only potent in the low micromolar regime, we next used AutoClickChem to identify ligands with even higher predicted binding energies. The same five alkyne analogues used previously were reacted *in silico* with the 1,215 azides used to generate the large virtual library. The 14,580 resulting products were again docked with Vina and rescored with the AutoDock 4.0 scoring function. In all, 214 compounds scored better than A13 (−11.07 kcal/mol). The best ligand (Figure 2) had a predicted binding energy of −13.33 kcal/mol.

The predicted binding pose of the best-scoring ligand is plausible (Figure 2). The 5-phenylisoxazole-3-carboxylic-acid portion of the ligand, first identified as a PTP1B inhibitor by researchers at Abbott Laboratories, was correctly positioned in the appropriate pocket as judged by x-ray crystallography [42]. This molecular fragment is predicted to participate in electrostatic, hydrogen-bond, and π-π stacking interactions with the protein receptor (Figure 2). The 1,2,3-azole ring is likewise predicted to participate in a hydrogen-bond interaction, as well as in a T-stacking interaction. Finally, the 2-nitrofuran azide fragment extends a nitro group near two arginine side chains, potentially facilitating additional receptor-ligand electrostatic interactions. A hydrogen bond with the furan oxygen atom is also predicted, further improving molecular recognition.

Interestingly, the top predicted ligand identified using AutoClickChem is similar to another ligand whose binding pose was recently characterized by x-ray crystallography (Figure 2, shown in purple) [41]. Both ligands span the same two pockets, and both position functional groups with negative charges (carboxylate, nitro, and sulfonate groups) at the same two locations.

In summary, we herein presented a computer algorithm called AutoClickChem that can simulate the reactions of click chemistry *in silico*. AutoClickChem can be used to generate large combinatorial libraries of easily synthesizable compound models for use in virtual screening. Additionally, the algorithm may prove useful in rational drug design and drug optimization. To demonstrate its utility, we used AutoClickChem to generate a large virtual library of easily synthesizable, drug-like, 1,2,3-azole compounds for use in virtual screens. Additionally, we reproduced two experimental applications of click-chemistry inhibitor optimization *in silico*.

We have also described the *pymolecule* toolbox, a python-based framework that facilitates the development of programs that require the manipulation of molecular models. Beta versions of *pymolecule* have been used to create a number of other useful python scripts; we are hopeful that the *pymolecule* toolbox, now well documented and consolidated into a single file (pymolecule.py), will be helpful to other computational chemists as well.

### Availability and Future Directions

While implementations of AutoClickChem and the *pymolecule* toolbox are available from the PLoS Computational Biology website, we recommend visiting http://autoclickchem.ucsd.edu to obtain the latest versions. Additionally, AutoClickChem has been implemented as an opal web service [43] and a server application at http://autoclickchem.ucsd.edu, enabling use without requiring installation.

The authors have plans to incorporate AutoClickChem into future projects as well. For example, the next generation of the AutoGrow algorithm [44] is currently being developed; among many improvements, the program will be extended using AutoClickChem. The original AutoGrow algorithm generated novel ligands by swapping hydrogen atoms with new molecular fragments. Unfortunately, this often produced molecular models of compounds that are difficult to synthesize. Newer versions of AutoGrow will add molecular fragments *via* the reactions of click chemistry, facilitating subsequent synthesis.

In time, we expect to add new features to *pymolecule* as well. Beta versions of the *pymolecule* toolbox have already been used in several projects; as new needs arise in the context of future projects, appropriate additions will be made to the public version of *pymolecule* as well.

We encourage others to modify the AutoClickChem and *pymolecule* source code. As both these resources are python implemented, extending the source code is not difficult. For example, users could extend AutoClickChem to include additional reactions. Some may also wish to expand the *pymolecule* toolbox by adding new functionality (*e.g.*, rmsd-alignment definitions, the ability to read formats other than PDB, etc.) as needs arise in their own projects. We encourage users to contact the authors with any significant modifications so they can be included in future versions of the software.

## Supporting Information

Figure S1 The click-chemistry reactions that can be simulated *in silico* using AutoClickChem.
(PDF)

Text S1 Contains additional details describing the *pymolecule* toolbox, the creation of the large virtual library of easily synthesizable compounds, and the docking protocol used in the current work. Further descriptions of each of the chemical reactions built into AutoClickChem are also provided, with extensive references.
(DOC)

Text S2 Compressed file of the AutoClickChem source code.
(TAR)

Text S3 Compressed file of the pymolecule source code.
(TAR)

Text S4 Compressed file of the AutoClickChem Rocks roll source code.
(TAR)
