# Supplementary material for: AutoClickChem: Click Chemistry in Silico
Source: PLoS Comput Biol. 2012 Mar 15;8(3):e1002397. doi: 10.1371/journal.pcbi.1002397 (PMC3305364; doi:10.1371/journal.pcbi.1002397)

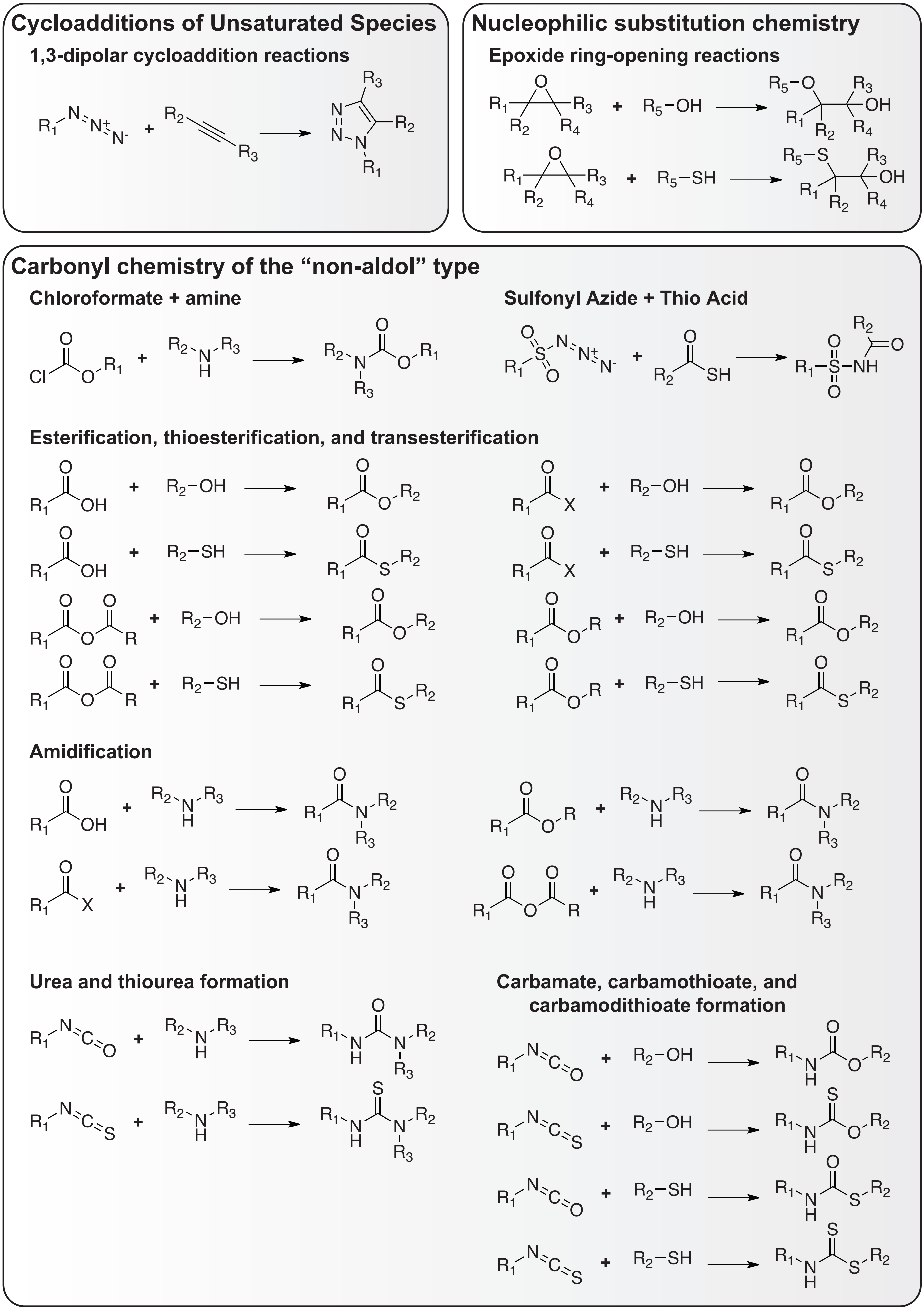

Cycloadditions of Unsaturated Species
1,3-dipolar cycloaddition reactions
Nucleophilic substitution chemistry
Epoxide ring-opening reactions
Carbonyl chemistry of the “non-aldol” type
Chloroformate + amine
Sulfonyl Azide + Thio Acid
Esterification, thioesterification, and transesterification
Amidification
Urea and thiourea formation
Carbamate, carbamothioate, and carbamodithioate formation

Additions to carbon-carbon multiple bonds

Epoxidation

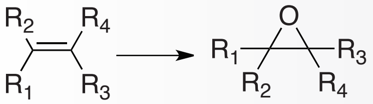

Supporting reactions

Azide and cyanide formation

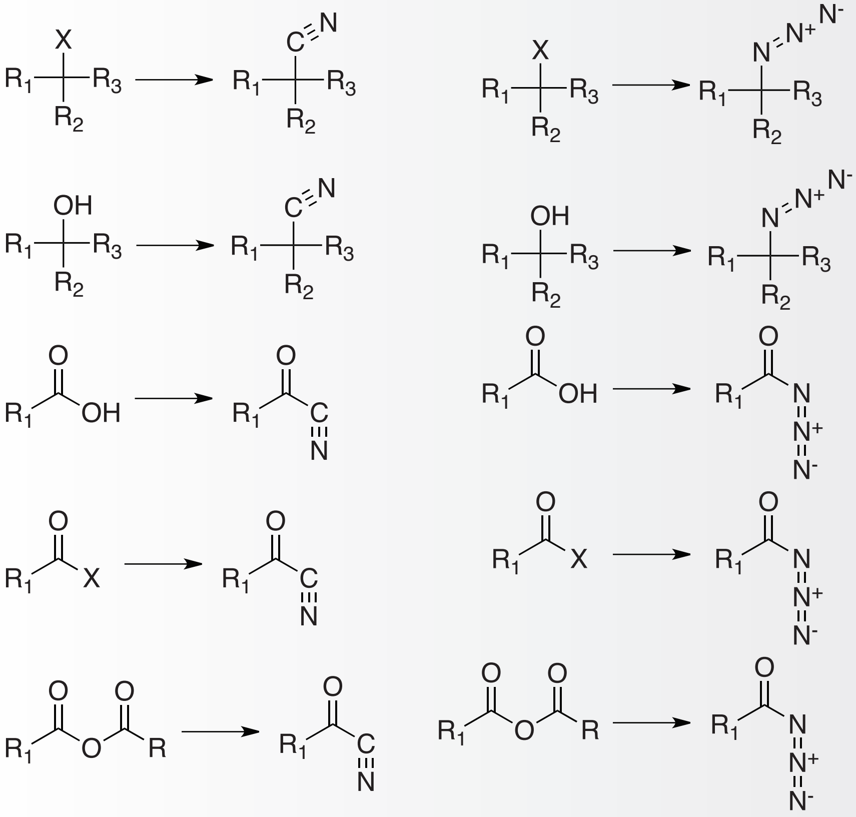

Primary amine oxidation and reduction to an amine

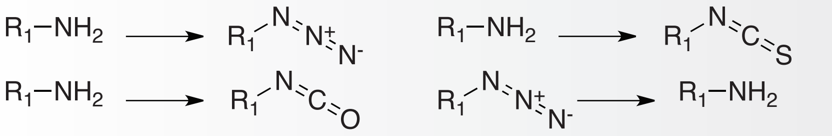

Supplement: Figure S1 — The click-chemistry reactions that can be simulated in silico using AutoClickChem. (PDF) [file pcbi.1002397.s001.pdf]
