# Supplementary material for: AutoClickChem: Click Chemistry in Silico
Source: PLoS Comput Biol. 2012 Mar 15;8(3):e1002397. doi: 10.1371/journal.pcbi.1002397 (PMC3305364; doi:10.1371/journal.pcbi.1002397)
Supplement: Text S1 — Contains additional details describing the pymolecule toolbox, the creation of the large virtual library of easily synthesizable compounds, and the docking protocol used in the current work. Further descriptions of each of the chemical reactions built into AutoClickChem are also provided, with extensive references. (DOC) [file pcbi.1002397.s002.doc]

**Experimental Details**

*The* pymolecule *Toolbox: Reaction-Product Assembly*

The AutoClickChem reaction engine is based on an open-source python toolbox that may be useful for other projects as well. The python definitions described in the main text can be used to identify reactive functional groups known to participate in click-chemistry reactions. In the current implementation of AutoClickChem, these functional groups include: azides, sulfonyl azides, thio acids, alkenes, alkynes, alcohols, thiols, epoxides, amines, carboxylates, acylhalides, esters, carbonochloridates, acid anhydrides, isocyanates, isothiocyanates, and halides.

The interested reader can examine the AutoClickChem source code to learn how a specific functional group is identified. As an example, consider the function used to detect azides, outlined in pseudo code below:

azides = a list
FOR each atom in the molecular model
    atom1 = the atom being considered
    IF atom1 is nitrogen AND IF atom1 is bound to one other atom THEN
         neighbor1 = the atom to which atom1 is bound
         IF neighbor1 is nitrogen AND IF neighbor1 is bound to two atoms THEN
              neighbor2 = the atom to which neighbor1 is bound (not atom1)
              IF neighbor2 is nitrogen AND IF neighbor2 is bound to two atoms THEN
                   IF angle atom1-neighbor1-neighbor2 is nearly linear THEN
                        neighbor3 = the atom to which neighbor2 is bound (not neighbor1)
                        azide_id = (atom1, neighbor1, neighbor2, neighbor3)
                        APPEND azide_id to azides
RETURN azides

Having identified a reactive group, these same functions can be used to simulate click-chemistry reactions *in silico*. The interested reader can again examine the AutoClickChem source code to learn how a specific reaction is performed. As an example, the pseudo code corresponding to the azide-alkyne Huisgen cycloaddition, also illustrated in Figure 1, should be helpful:

azide_PDB = molecular model containing an azide
alkyne_PDB = model containing an alkyne

azide_atom1 = azide distal nitrogen
azide_atom2 = azide medial nitrogen
azide_atom3 = azide proximal nitrogen
azide_atom4 = carbon bound to azide_atom3

alkyne_atom1 = one of the triple-bonded carbons
alkyne_atom2 = carbon single-bonded to alkyne_atom1
alkyne_atom3 = the other triple-bonded carbon
alkyne_atom4 = carbon single-bonded to alkyne_atom3

alkyne_fragment1 = the fragment with alkyne_atom1 after the alkyne_PDB triple bond is cut
alkyne_fragment2 = the other fragment obtained by cutting

intermediate_PDB = the intermediate molecular structure shown in Figure 1C

tethers1 = two intermediate atoms connected to alkyne_atom1 and alkyne_atom2 (Figure 1C)
MINIMIZE the lengths of these tethers by translating and rotating alkyne_fragment1

tethers2 = two intermediate atoms connected to alkyne_atom3 and alkyne_atom4 (Figure 1C)
MINIMIZE the lengths of these tethers by translating and rotating alkyne_fragment2

tethers3 = two intermediate atoms connected to azide_atom3 and azide_atom4 (Figure 1C)
MINIMIZE the lengths of these tethers by translating and rotating azide_PDB

DELETE atoms from alkyne_fragment1, alkyne_fragment2, azide_PDB, and intermediate_PDB to eliminate overlapping and obsolete atoms

ROTATE alkyne_fragment1, alkyne_fragment2, and azide_PDB around bonds that connect them to intermediate_PDB to minimize steric clashes

merged_PDB = alkyne_fragment1, alkyne_fragment2, azide_PDB, and intermediate_PDB merged into a single molecular model

RETURN merged

*Generating a Large Virtual Library of Easily Synthesizable Compounds*

To show how AutoClickChem can be used to generate a large virtual library of compounds that can be easily synthesized *in vitro* using the reactions of click chemistry, a virtual library of roughly 800,000 1,2,3-triazole compounds was created. First, 2,161 unique compounds containing terminal alkynes were identified from among the compounds available from hit2lead.com. These compounds were downloaded and processed using the Schodinger program LigPrep (Schodinger). When the multiple charged, tautomeric, ring-conformational, and stereoisomeric states of these compounds were considered, 3,690 small-molecule models were produced. Finally, models that had associated molecular weights greater than 300 daltons or that contained multiple alkynes were discarded; 939 alkyne models remained.

A similar protocol was used to generate azide models. 9,402 unique alkyl bromides, vinyl bromides, and aromatic bromides were identified from among those compounds available commercially from hit2lead.com. These compounds were also downloaded and processed using LigPrep (Schodinger). When multiple charged, tautomeric, ring-conformational, and stereoisomeric states were considered, 13,226 bromide models were produced. Those models with molecular weight greater than 300, as well as those with multiple bromides, were discarded; 1,220 models remained. AutoClickChem was used to generate 1,215 azides from these 1,220 bromide models .

AutoClickChem was again used, this time to combine the 939 alkyne with the 1,215 azide models in order to produce 2,281,770 1,2,3-triazole models that could be easily synthesized *in vitro* *via* the azide-alkyne Huisgen cycloaddition reaction . Of these AutoClickChem-generated models, 667 had steric clashes and were removed. The remaining compounds were filtered for Lipinski’s rule-of-five criteria using Open Babel and some in-house scripts. Any compound found to violate any of Lipinski’s criteria was discarded. We note that this is stricter than Lipinski’s original requirements, which permit one violation. Finally, the geometries of the roughly 800,000 remaining compounds were optimized using the *obminimize* program distributed with the Open Babel package .

*Computer Docking*

In order to demonstrate the utility of AutoClickChem, AutoClickChem-generated compounds were docked into crystal structures of acetylcholinesterase (AChE, PDB ID: 1Q83) and protein tyrosine phosphatase 1B (PTP1B , PDB ID: 2F71) . Hydrogen atoms were added to the protein receptors using PDB2PQR , and atom types and Gasteiger partial atomic charges were assigned using MGL Tools (ADT) .

To generate potential AChE inhibitors, the 23 alkynes and the tacrine-like azide used by Krasinski et al. were recreated *in silico* using Schrodinger Maestro (Schrodinger). The Schrodinger program LigPrep was then used to generate the alternate charged, tautomeric, ring-conformational, and stereoisomeric states of each of these fragments, producing 118 alkyne and 6 azide models. AutoClickChem was then used to react each of these alkynes with each of the azides. As both the 1,4 and 1,5 regioisomers were possible products, a total of 1,416 1,2,3-triazole models were generated.

To generate potential PTP1B inhibitors, the 14 azides and the 5 alkynes used by Srinivasan et al. were recreated *in silico*, as above. When these molecular fragments were processed with LigPrep, 6 alkyne and 18 azide models resulted. As Srinivasan et al. used a copper (I) catalyst, only the 1,4-regioisomer was produced . Consequently, AutoClickChem was used to generate only 108 1,4-adduct products. A second round of potential PTP1B inhibitors was generated by reacting the same 6 alkyne models with the 1,215 azide models used to generate the large virtual library.

AutoDock Vina was used to dock these AutoClickChem-generated compounds into their respective receptors. Atom types and Gasteiger partial atomic charges were assigned to all ligands using MGL Tools (ADT) , and the compounds were docked into boxes encompassing the respective protein active sites. All Vina-docked poses were rescored using the AutoDock 4.0 scoring function without redocking , ignoring the energy of the unbound state. Each Vina docking suggested several possible poses. Each of these poses was evaluated using the AutoDock 4.0 scoring function, and the score associated with the best AutoDock score, not necessarily the same pose as that associated with the best Vina score, was used in subsequent ranking.

As the LigPrep program was used to generate redundant molecular fragments with differing charged, tautomeric, ring-conformational, and stereoisomeric states, there were multiple versions of each AutoClickChem-generated compound. In order to facilitate comparison with experimental results, redundant compounds were eliminated from the ranked lists. The AutoDock score ultimately assigned to each unique compound was the best score associated with any of its forms.

**AutoClickChem Reactions**

The “click” reactions can be divided into four general classes : cycloadditions of unsaturated species, nucleophilic substitution chemistry, “non-aldol” carbonyl chemistry, and additions to carbon-carbon multiple bonds.

*Cycloadditions of Unsaturated Species*

As explained in the Materials and Methods, AutoClickChem can mimic the azide-alkyne Huisgen cycloaddition, wherein an alkyne and an azide react to yield a 1,2,3-triazole product (Figures 1 and S1) . AutoClickChem creates both the 1,4 and the 1,5 regioisomers. *In vitro*, catalysts can be used to select one regioisomer over the other. For example, the use of a copper (I) catalyst favors the 1,4 adduct .

*Nucleophilic Substitution Chemistry*

AutoClickChem can also mimic an epoxide ring-opening reaction (Figure S1) . The non-ideal bond angles of an epoxide ring are highly strained and therefore susceptible to opening by nucleophiles. AutoClickChem can mimic epoxide opening by alcohols and thiols and can generate all regioisomers. *In vitro*, reaction conditions can again be used to control regiospecificity .

*“Non-Aldol” Carbonyl Chemistry*

Many reactions involving carbonyl functional groups can be considered “click.” The electronegative carbonyl oxygen atom draws electrons away from the carbon atom, making that atom more susceptible to nucleophilic attack. AutoClickChem is capable of performing a number of carbonyl reactions *in silico*.

Chloroformates can react with amines to produce carbamates (Figure S1) . The chloride atom is a good leaving group, leaving the carbonyl carbon atom susceptible to nucleophilic attack by the amine. AutoClickChem generates both the *cis* and *trans* stereoisomers. Additionally, AutoClickChem is also capable of performing the “sulfo-click” reaction *in silico*, in which a sulfonyl azide reacts with a thio acid to produce an acyl sulfonamide (Figure S1) .

A number of esterfication, thioesterification, and transesterification reactions are also useful. AutoClickChem can generate ester models from models of alcohols and acyl halides (essentially mimicking the Schotten–Baumann method) ; carboxylates (i.e., alkoxy-de-hydroxylation) ; anhydrides (where both products are produced) ; and other esters (i.e., transesterification) . AutoClickChem also permits the use of thiols in place of alcohols for all esterification and transesterification reactions. Both *cis* and *trans* amides can likewise be produced by reacting amines with carboxylates, acyl halides, esters, and anhydrides *in silico*, again mimicking the Schotten–Baumann method (Figure S1) .

AutoClickChem can also form ureas and thioureas by reacting isocyanates and isothiocyanates with amines *via N*-hydro-*C*-alkylamino-addition (Figure S1) . Again, both the *cis* and *trans* products are formed. Likewise, carbamates, carbamothioates, and carbamodithioates can be formed by reacting isocyanates and isothiocyanates with alcohols and thiols *via N*-hydro-*C*-alkoxy-addition (Figure S1) .

*Additions to Carbon-Carbon Multiple Bonds*

AutoClickChem can also simulate epoxidation *in silico*, converting a carbon-carbon double bond into a reactive epoxide with a strained three-member ring . As described above, epoxide rings are highly susceptible to nucleophilic opening. After constructing epoxide models, AutoClickChem can be applied a second time to open these epoxides with nucleophilic alcohols and thiols.

*Supporting Reactions*

AutoClickChem is also capable of mimicking a number of reactions needed to create the azides, isocyanates, and isothiocyanates so often used in click chemistry. Azides can be generated from alcohols , halides , carboxylic acids , acyl halides , and anhydrides . AutoClickChem is also capable of generating cyanides from these reagents . Additionally, amines can be oxidized to azides , isocyanates, and isothiocyanates . For completeness, AutoClickChem can also reduce an azide to an amine .

**References**

1. Scriven EFV, Turnbull K (1988) Azides: their preparation and synthetic uses. Chemical Reviews 88: 297-368.

2. Alvarez SG, Alvarez MT (1997) A practical procedure for the synthesis of alkyl azides at ambient temperature in dimethyl sulfoxide in high purity and yield Synthesis 4: 413-414.

3. Miller JA (1975) Synthesis of tertiary alkyl azides Tetrahedron Letters 16: 2959-2960.

4. Huisgen R. Centenary Lecture - 1,3-Dipolar Cycloadditions; 1961. pp. 357-396.

5. Lipinski CA, Lombardo F, Dominy BW, Feeney PJ (2001) Experimental and computational approaches to estimate solubility and permeability in drug discovery and development settings. Adv Drug Deliv Rev 46: 3-26.

6. Guha R, Howard MT, Hutchison GR, Murray-Rust P, Rzepa H, et al. (2006) The Blue Obelisk-interoperability in chemical informatics. J Chem Inf Model 46: 991-998.

7. Bourne Y, Kolb HC, Radic Z, Sharpless KB, Taylor P, et al. (2004) Freeze-frame inhibitor captures acetylcholinesterase in a unique conformation. Proc Natl Acad Sci U S A 101: 1449-1454.

8. Klopfenstein SR, Evdokimov AG, Colson AO, Fairweather NT, Neuman JJ, et al. (2006) 1,2,3,4-Tetrahydroisoquinolinyl sulfamic acids as phosphatase PTP1B inhibitors. Bioorg Med Chem Lett 16: 1574-1578.

9. Dolinsky TJ, Czodrowski P, Li H, Nielsen JE, Jensen JH, et al. (2007) PDB2PQR: expanding and upgrading automated preparation of biomolecular structures for molecular simulations. Nucleic Acids Res 35: W522-W525.

10. Dolinsky TJ, Nielsen JE, McCammon JA, Baker NA (2004) PDB2PQR: an automated pipeline for the setup of Poisson-Boltzmann electrostatics calculations. Nucleic Acids Res 32: W665-W667.

11. Gasteiger J, Marsili M (1980) Iterative partial equalization of orbital electronegativity--a rapid access to atomic charges. Tetrahedron 36: 3219-3228.

12. Sanner MF (2005) A component-based software environment for visualizing large macromolecular assemblies. Structure 13: 447-462.

13. Krasinski A, Radic Z, Manetsch R, Raushel J, Taylor P, et al. (2005) In situ selection of lead compounds by click chemistry: target-guided optimization of acetylcholinesterase inhibitors. J Am Chem Soc 127: 6686-6692.

14. Srinivasan R, Uttamchandani M, Yao SQ (2006) Rapid assembly and in situ screening of bidentate inhibitors of protein tyrosine phosphatases. Org Lett 8: 713-716.

15. Tornøe CW, Christensen C, Meldal M (2002) Peptidotriazoles on Solid Phase: [1,2,3]-Triazoles by Regiospecific Copper(I)-Catalyzed 1,3-Dipolar Cycloadditions of Terminal Alkynes to Azides. Journal of Organic Chemistry 67: 3057-3064.

16. Trott O, Olson AJ (2009) AutoDock Vina: Improving the speed and accuracy of docking with a new scoring function, efficient optimization, and multithreading. J Comput Chem 31: 455-461.

17. Morris GM, Goodsell DS, Halliday RS, Huey R, Hart WE, et al. (1998) Automated docking using a Lamarckian genetic algorithm and an empirical binding free energy function. Journal of Computational Chemistry 19: 1639-1662.

18. Kolb HC, Finn MG, Sharpless KB (2001) Click Chemistry: Diverse Chemical Function from a Few Good Reactions. Angew Chem Int Ed Engl 40: 2004-2021.

19. Banert K (1989) Reactions of Unsaturated Azides, 6. Synthesis of 1,2,3-Triazoles from Propargyl Azides by Rearrangement of the Azido Group. – Indication of Short-Lived Allenyl Azides and Triazafulvenes. Chemische Berichte 122: 911–918.

20. Gothelf KV, Jorgensen KA (1998) Asymmetric 1,3-Dipolar Cycloaddition Reactions. Chem Rev 98: 863-910.

21. Fringuelli F, Piermatti O, Pizzo F, Vaccaro L (1999) Ring Opening of Epoxides with Sodium Azide in Water. A Regioselective pH-Controlled Reaction. J Org Chem 64: 6094–6096.

22. Mormeneo D, Llebaria A, Delgado A (2004) A practical synthesis of carbamates using an 'in-situ' generated polymer-supported chloroformate. Tetrahedron Letters 45: 6831-6834.

23. Rijkers DTS, Merkx R, Yim CB, Brouwer AJ, Liskamp RMJ (2010) 'Sulfo-click' for ligation as well as for site-specific conjugation with peptides, fluorophores, and metal chelators. Journal of Peptide Science 16: 1-5.

24. Baumann E (1886) Ueber eine einfache Methode der Darstellung von Benzoësäureäthern. Berichte der deutschen chemischen Gesellschaft 19: 3218-3222.

25. Haslam E (1980) Recent developments in methods for the esterification and protection of the carboxyl group. Tetrahedron 36: 2409-2433.

26. Procopiou PA, Baugh SPD, Flack SS, Inglis GGA (1996) An extremely fast and efficient acylation reaction of alcohols with acid anhydrides in the presence of trimethylsilyl trifluoromethanesulfonate as catalyst Chemical Communications 23: 2625-2626.

27. Otera J (1993) Transesterification. Chemical Reviews 93: 1449-1470.

28. Schotten C (1884) Ueber die Oxydation des Piperidins. Berichte der deutschen chemischen Gesellschaft 17: 2544-2547.

29. Satchell DPN, Satchell RS (1975) Acylation by ketens and isocyanates. A mechanistic comparison. Chem Soc Rev 4: 231-250.

30. Vishnyakova TP, Golubeva IA, Glebova EV (1985) Substituted Ureas. Methods of Synthesis and Applications Russian Chemical Reviews 54: 249.

31. Herr RJ, Kuhler JL, Meckler H, Opalka CJ (2000) A Convenient Method for the Preparation of Primary and Symmetrical N,N′-Disubstituted Thioureas. Synthesis 11: 1569-1574.

32. Kim YH, Park HS (1998) A convenient coupling reaction of isocyanates with t-alcohols with catalytic amount of SmI2 in the presence of HMPA. Synlett: 261-262.

33. Hazzard G, Lammiman SA, Poon NL, Satchell DPN, Satchell RS (1985) The Kinetics of the Addition of Ethanol to Para-Chlorophenyl Isocyanate in Diethyl-Ether Solution in the Presence of Covalent Metal-Halides. Journal of the Chemical Society-Perkin Transactions 2: 1029-1033.

34. Duggan ME, Imagire JS (1989) Copper(I) Chloride Catalyzed Addition of Alcohols to Alkyl Isocyanates - a Mild and Expedient Method for Alkyl Carbamate Formation. Synthesis-Stuttgart: 131-132.

35. Mcmanus SP, Bruner HS, Coble HD, Ortiz M (1977) Studies of Catalyzed Reaction between Alcohols and Alkyl Isocyanates - Evidence for a Light-Assisted Reaction. Journal of Organic Chemistry 42: 1428-1433.

36. Bailey WJ, Griffith JR (1978) Convenient Synthesis of Tertiary Alkyl N-Phenylcarbamates from Tertiary Alcohols and Phenyl Isocyanate with a Lithium Alkoxide Catalyst. Journal of Organic Chemistry 43: 2690-2692.

37. Nikiforov A, Jirovetzb L, Buchbauer G (1989) Synthesis of Tertiary Alcohol Carbarnates Liebigs Annalen der Chemie 1989: 489-491.

38. Smith M, March J (2007) Chapter 16: Addition to Carbon-Hetero Multiple Bonds. March's advanced organic chemistry : reactions, mechanisms, and structure. 6th ed. Hoboken, N.J.: Wiley-Interscience. pp. xx, 2357 p.

39. Herrmann WA, Fischer RW, Marz DW (2003) Methyltrioxorhenium as Catalyst for Olefin Oxidation. Angewandte Chemie International Edition in English 30: 1638-1641.

40. Adolfsson H, Converso A, Sharpless KB (1999) Comparison of amine additives most effective in the new methyltrioxorhenium-catalyzed epoxidation process Tetrahedron Letters 40: 3991-3994.

41. Herrmann WA, Kühn FE (1997) Organorhenium Oxides. Accounts of Chemical Research 30: 169-180.

42. Rudolph J, Reddy KL, Chiang JP, Sharpless KB (1997) Highly Efficient Epoxidation of Olefins Using Aqueous H2O2 and Catalytic Methyltrioxorhenium/Pyridine: Pyridine-Mediated Ligand Acceleration. Journal of the American Chemical Society 119: 6189–6190.

43. Thompson AS, Humphrey GR, DeMarco AM, Mathre DJ, Grabowski EJJ (1993) Direct conversion of activated alcohols to azides using diphenyl phosphorazidate. A practical alternative to Mitsunobu conditions. Journal of Organic Chemistry 58: 5886–5888.

44. Mitsunobu O, Yamada M (1967) Preparation of Esters of Carboxylic and Phosphoric Acid via Quaternary Phosphonium Salts. Bulletin of the Chemical Society of Japan 40: 2380-2382.

45. Kumar HMS, Reddy BVS, Anjaneyulu S, Yadav JS (1998) An expedient and highly selective conversion of alcohols to azides using a NaN3/BF3·Et2O system Tetrahedron Letters 39: 7385-7388

46. Adam G, Andrieux J, Plat M (1985) Decomposition acido-catalysee d'azides tertiaires benzocyclobuteniques. Nouvelle methode de synthese du noyau indolique par extension du cycle Tetrahedron 41: 399-407.

47. Hassner A, Fibiger R, Andisik D (1984) Synthetic methods. 19. Lewis acid catalyzed conversion of alkenes and alcohols to azides. J Org Chem 49: 4237-4244.

48. Balderman D, Kalir A (1978) Selective Reduction of Azides. Improved Preparation of α,α-Disubstituted Benzylamines. Synthesis 1: 24-26.

49. Lwowski W (1971) In: Patai S, editor. The Chemistry of the Azido Group. New York: Wiley Interscience. pp. 503-554.

50. Camps F, Gasol V, Guerrero A (1988) A Simple One-Pot Synthesis of Nitriles from Alcohols. Synthetic Communications 18: 445-452.

51. Manna S, Falck J, Mioskowski C (1985) A convenient preparation of alkyl halides and cyanides from alcohols by modification of the Mitsunobu procedure. Synthetic Communications 15: 663-668.

52. Mizuno A, Hamada Y, Shioiri T (1980) New Methods and Reagents in Organic Synthesis; A Simple One-Pot Conversion of Primary Alcohols to Nitriles having One C-Atom more. Synthesis 12: 1007-1009.

53. Davis R, Untch KG (1981) Direct one-step conversion of alcohols into nitriles. Journal of Organic Chemistry 46: 2985-2987.

54. Smiley R, Arnold C (1960) Notes- Aliphatic Nitriles from Alkyl Chlorides. Journal of Organic Chemistry 25: 257-258.

55. Friedman L, Shechter H (1960) Preparation of Nitriles from Halides and Sodium Cyanide. An Advantageous Nucleophilic Displacement in Dimethyl Sulfoxide. Journal of Organic Chemistry 25: 877-879.

56. Hünig S, Schaller R (1982) The Chemistry of Acyl Cyanides. Angewandte Chemie International Edition in English 21: 36-49.

57. Reddy GVS, Rao GV, Subramanyam RVK, Iyengar DS (2000) A New Novel and Practical One Pot Methodology for Conversion of Alcohols to Amines. Synthetic Communications 30: 2233-2237.

58. Sigurdsson ST, Seeger B, Kutzke U, Eckstein F (1995) A Mild and Simple Method for the Preparation of Isocyanates from Aliphatic Amines Using Trichloromethyl Chloroformate. Synthesis of an Isocyanate Containing an Activated Disulfide. J Org Chem 61: 3883-3884.

59. Rolla F (1982) Sodium borohydride reactions under phase-transfer conditions: reduction of azides to amines. The Journal of Organic Chemistry 47: 4327-4329.

60. Larock RC (1999) Comprehensive Organic Transformations, 2nd ed. New York: Wiley-VCH.
