# Supplementary material for: AutoClickChem: Click Chemistry in Silico
Source: PLoS Comput Biol. 2012 Mar 15;8(3):e1002397. doi: 10.1371/journal.pcbi.1002397 (PMC3305364; doi:10.1371/journal.pcbi.1002397)
Supplement: Text S3 — Compressed file of the pymolecule source code. (TAR) [file pcbi.1002397.s004.tar › AutoClickChem_rocks_roll_1_0_0/src/autoclickchem-web/autoclickchem-web-1.0.0.tar.bz2!/autoclickchem-web-1.0.0/help/zip_file.php]

AutoClickChem Download


Upload a library of compounds. The uploaded file should be a *zip* file containing 3-D models of the ligands in one of the specified formats (*pdb* preferred). The *zip* file should not include any subdirectories, and each compound should be present in its own file.
