# Supplementary material for: AutoClickChem: Click Chemistry in Silico
Source: PLoS Comput Biol. 2012 Mar 15;8(3):e1002397. doi: 10.1371/journal.pcbi.1002397 (PMC3305364; doi:10.1371/journal.pcbi.1002397)
Supplement: Text S3 — Compressed file of the pymolecule source code. (TAR) [file pcbi.1002397.s004.tar › AutoClickChem_rocks_roll_1_0_0/src/autoclickchem-web/autoclickchem-web-1.0.0.tar.bz2!/autoclickchem-web-1.0.0/help/what_does_autoclick_do.php]

What Does AutoClickChem do?


Virtual screening is a valuable way to identify novel protein inhibitors. Docking software is used to "predict how small molecules, such as substrates or drug candidates, bind to a [protein] receptor of known 3D structure." Especially when coupled with advanced docking techniques like the Relaxed Complex Scheme, computer docking can be quite predictive, but experimental validation is always essential.

Experimental validation requires that the predicted inhibitors be chemically available. Thus one of the following criteria must be satisfied:

1. The predicted inhibitors must be obtainable from educational, governmental, or commercial chemical libraries
2. The chemical synthesis of the predicted inhbitors must be feasible.

Because synthesis allows for greater compound diversity, many computational chemists design molecules from scratch. However, computational chemists are notorious for designing compounds that cannot be easily synthesized.

**AutoClickChem** carries out synthesis *in silico* according to the rules of click chemistry. Because AutoClickChem only produces compounds that can be easily synthesized by synthetic chemists, it can greatly accelerate the drug-discovery process. Virtual screening can be carried out on AutoClickChem-generated compound libraries to identify potential protein inhibitors. The predicted inhibitors can be easily synthesized *via* click chemistry, making experimental validation fast and simple.
