# Supplementary material for: AutoClickChem: Click Chemistry in Silico
Source: PLoS Comput Biol. 2012 Mar 15;8(3):e1002397. doi: 10.1371/journal.pcbi.1002397 (PMC3305364; doi:10.1371/journal.pcbi.1002397)
Supplement: Text S3 — Compressed file of the pymolecule source code. (TAR) [file pcbi.1002397.s004.tar › AutoClickChem_rocks_roll_1_0_0/src/autoclickchem-web/autoclickchem-web-1.0.0.tar.bz2!/autoclickchem-web-1.0.0/help/starting_library.php]

Chemical Libraries


AutoClickChem requires two libraries (groups) of small-molecule models. To create chemical products derived according to the rules of click chemistry, AutoClickChem selects compounds from the first library and reacts them with compounds from the second library.

One strategy is to populate the first library with known protein inhibitors. The second library is then populated with small fragments that can react with the known inhibitors according to the rules of click chemistry. AutoClickChem can then be used to generate a library of novel compounds with potentially improved binding affinities, as assessed by a docking program.
