# Supplementary material for: AutoClickChem: Click Chemistry in Silico
Source: PLoS Comput Biol. 2012 Mar 15;8(3):e1002397. doi: 10.1371/journal.pcbi.1002397 (PMC3305364; doi:10.1371/journal.pcbi.1002397)
Supplement: Text S3 — Compressed file of the pymolecule source code. (TAR) [file pcbi.1002397.s004.tar › AutoClickChem_rocks_roll_1_0_0/src/autoclickchem-web/autoclickchem-web-1.0.0.tar.bz2!/autoclickchem-web-1.0.0/help/ligand_size.php]

Ligand Size


The user can tell AutoClickChem to avoid generating products that are larger than a given molecular weight or that contain more than a specified maximum number of atoms. There's good reason to limit the size of potential lead compounds; Christopher Lipinski conducted a study in 1997 wherein he determined that most orally-available drugs have molecular weights less than 500 daltons. Lead compounds should probably be even less massive, allowing for fragment additions later on in the drug-development process.
