# Supplementary material for: AutoClickChem: Click Chemistry in Silico
Source: PLoS Comput Biol. 2012 Mar 15;8(3):e1002397. doi: 10.1371/journal.pcbi.1002397 (PMC3305364; doi:10.1371/journal.pcbi.1002397)
Supplement: Text S3 — Compressed file of the pymolecule source code. (TAR) [file pcbi.1002397.s004.tar › AutoClickChem_rocks_roll_1_0_0/src/autoclickchem-web/autoclickchem-web-1.0.0.tar.bz2!/autoclickchem-web-1.0.0/help/reaction_types.php]

Reaction Types


AutoClickChem performs the chemical reactions of click chemistry *in silico*. The user can specify which chemical reactions to perform.

Click chemistry includes chemical reactions that satisfy the following criteria:

1. application modular and wide in scope
2. obtains high chemical yield
3. generates inoffensive byproducts
4. is stereospecific
5. simple reaction conditions
6. has readily available starting materials and reagents
7. no solvent involved or a benign solvent (preferably water)
8. easy product isolation by crystallisation or distillation but not preparative chromatography
9. physiologically stable
10. large thermodynamic driving force > 84 kJ/mol to favor a reaction with a single reaction product. A distinct exothermic reaction makes a reactant "spring loaded".
11. high atom economy
