# Supplementary material for: AutoClickChem: Click Chemistry in Silico
Source: PLoS Comput Biol. 2012 Mar 15;8(3):e1002397. doi: 10.1371/journal.pcbi.1002397 (PMC3305364; doi:10.1371/journal.pcbi.1002397)
Supplement: Text S3 — Compressed file of the pymolecule source code. (TAR) [file pcbi.1002397.s004.tar › AutoClickChem_rocks_roll_1_0_0/src/autoclickchem-web/autoclickchem-web-1.0.0.tar.bz2!/autoclickchem-web-1.0.0/help/reactions.php]

AutoClickChem Reactions


AutoClickChem can perform a number of click-chemistry reactions. Please specify which types of reactions you with to perform below.
