# Supplementary material for: AutoClickChem: Click Chemistry in Silico
Source: PLoS Comput Biol. 2012 Mar 15;8(3):e1002397. doi: 10.1371/journal.pcbi.1002397 (PMC3305364; doi:10.1371/journal.pcbi.1002397)
Supplement: Text S3 — Compressed file of the pymolecule source code. (TAR) [file pcbi.1002397.s004.tar › AutoClickChem_rocks_roll_1_0_0/src/autoclickchem-web/autoclickchem-web-1.0.0.tar.bz2!/autoclickchem-web-1.0.0/help/multi-step.php]

Multi-Step Reactions


This feature allows AutoClickChem to add predicted products to the list of possible reactants, thus permitting multi-step reactions and greater product diversity.
