# Supplementary material for: AutoClickChem: Click Chemistry in Silico
Source: PLoS Comput Biol. 2012 Mar 15;8(3):e1002397. doi: 10.1371/journal.pcbi.1002397 (PMC3305364; doi:10.1371/journal.pcbi.1002397)
Supplement: Text S3 — Compressed file of the pymolecule source code. (TAR) [file pcbi.1002397.s004.tar › AutoClickChem_rocks_roll_1_0_0/src/autoclickchem-web/autoclickchem-web-1.0.0.tar.bz2!/autoclickchem-web-1.0.0/help/delete_duplicates.php]

Delete Duplicates


When selected, Autoclick will attempt to delete duplicate products. Each product is converted into a cannonical SMILES string, and SMILES strings are compared to identify duplicate products. Unfortunately, sometimes distinct molcules (i.e. the *cis* and *trans* conformations of amide bonds) have the same SMILES string. Because duplicate products are unlikely, AutoClickChem does not attempt to delete duplicates by default.
