# Supplementary material for: AutoClickChem: Click Chemistry in Silico
Source: PLoS Comput Biol. 2012 Mar 15;8(3):e1002397. doi: 10.1371/journal.pcbi.1002397 (PMC3305364; doi:10.1371/journal.pcbi.1002397)
Supplement: Text S3 — Compressed file of the pymolecule source code. (TAR) [file pcbi.1002397.s004.tar › AutoClickChem_rocks_roll_1_0_0/src/autoclickchem-web/autoclickchem-web-1.0.0.tar.bz2!/autoclickchem-web-1.0.0/help/click_chemistry.php]

Click Chemistry


"Click chemistry" is a chemical philosophy introduced by K. Barry Sharpless in 2001 and describes chemistry tailored to generate substances quickly and reliably by joining small units together. This is inspired by the fact that nature also generates substances by joining small modular units.

In biochemistry, proteins are made from repeating amino acid units and sugars are made from repeating monosaccharide units. The connecting units are based on carbon-hetero atom bonds C-X-C rather than carbon-carbon bonds. In addition, enzymes ensure that chemical processes can overcome large enthalpy hurdles by division into a series of reactions each with a small energy step. Mimicking nature in organic synthesis of new pharmaceuticals is essential given the large number of possible structures.

In 1996 Guida calculated the size of the pool of drug candidates at 10^63^, based on the presumption that a candidate consists of less than 30 non-hydrogen atoms, weighs less than 500 daltons, is made up of atoms of hydrogen, carbon, nitrogen, oxygen, phosphorus, sulfur, chlorine and bromine, and is stable at room temperature and stable towards oxygen and water. Click chemistry in combination with combinatorial chemistry, high-throughput screening and building chemical libraries speeds up new drug discoveries by making each reaction in a multistep synthesis fast, efficient and predictable.

Click chemistry encourages the following criteria:

- application modular and wide in scope
- obtains high chemical yield
- generates inoffensive byproducts
- is stereospecific
- simple reaction conditions
- has readily available starting materials and reagents
- no solvent involved or a benign solvent (preferably water)
- easy product isolation by crystallisation or distillation but not preparative chromatography
- physiologically stable
- large thermodynamic driving force > 84 kJ/mol to favor a reaction with a single reaction product. A distinct exothermic reaction makes a reactant "spring loaded".
- high atom economy

Many of the criteria are subjective; and even if measurable and objective criteria could be agreed upon, it's unlikely that any reaction will be perfect for every situation and application. However, several reactions have been identified which fit the bill better than others:

- the Huisgen 1,3-dipolar cycloaddition, in particular the Cu(I)-catalyzed stepwise variant, is often referred to simply as the "click reaction". Fokin and Sharpless independently described it as a reliable catalytic process offering "an unprecedented level of selectivity, reliability, and scope for those organic synthesis endeavors which depend on the creation of covalent links between diverse building blocks," firmly placing it among the most reliable processes fitting the click criteria.
- other cycloadditions such as the Diels-Alder reaction
- nucleophilic substitution especially to small strained rings like epoxy and aziridine compounds
- carbonyl-chemistry-like formation of ureas but not reactions of the aldol type due to low thermodynamic driving force.
- addition reactions to carbon-carbon double bonds like dihydroxylation.

(Adapted from Wikipedia.)
