# Supplementary material for: AutoClickChem: Click Chemistry in Silico
Source: PLoS Comput Biol. 2012 Mar 15;8(3):e1002397. doi: 10.1371/journal.pcbi.1002397 (PMC3305364; doi:10.1371/journal.pcbi.1002397)
Supplement: Text S3 — Compressed file of the pymolecule source code. (TAR) [file pcbi.1002397.s004.tar › AutoClickChem_rocks_roll_1_0_0/src/autoclickchem-web/autoclickchem-web-1.0.0.tar.bz2!/autoclickchem-web-1.0.0/help/num_products.php]

Number of Products


AutoClickChem performs *in silico* reactions by combining a small-molecule model from the first library with a small-molecule model from the second library according to the reactions of click chemistry. AutoClickChem will keep reacting pairs of compounds until the user-specified number of pairs has been reached. The online version of AutoClickChem allows at most 100 pairs. If you'd like to perform more reactions, please download a free copy of AutoClickChem to use on your own machine.
